# High accuracy mass spectrometry analysis as a tool to verify and improve gene annotation using *Mycobacterium tuberculosis *as an example

**DOI:** 10.1186/1471-2164-9-316

**Published:** 2008-07-02

**Authors:** Gustavo A de Souza, Hiwa Målen, Tina Søfteland, Gisle Sælensminde, Swati Prasad, Inge Jonassen, Harald G Wiker

**Affiliations:** 1 Section for Microbiology and Immunology, The Gade Institute, University of Bergen, Bergen, Norway; 2 Department of Informatics, High Technology Center, University of Bergen, Bergen, Norway; 3 Computational Biology Unit, BCCS, High Technology University of Bergen, Bergen, Norway; 4 Department of Microbiology and Immunology, Haukeland University Hospital, Bergen, Norway

## Abstract

**Background:**

While the genomic annotations of diverse lineages of the *Mycobacterium tuberculosis *complex are available, divergences between gene prediction methods are still a challenge for unbiased protein dataset generation. *M. tuberculosis *gene annotation is an example, where the most used datasets from two independent institutions (Sanger Institute and Institute of Genomic Research-TIGR) differ up to 12% in the number of annotated open reading frames, and 46% of the genes contained in both annotations have different start codons. Such differences emphasize the importance of the identification of the sequence of protein products to validate each gene annotation including its sequence coding area.

**Results:**

With this objective, we submitted a culture filtrate sample from *M. tuberculosis *to a high-accuracy LTQ-Orbitrap mass spectrometer analysis and applied refined N-terminal prediction to perform comparison of two gene annotations. From a total of 449 proteins identified from the MS data, we validated 35 tryptic peptides that were specific to one of the two datasets, representing 24 different proteins. From those, 5 proteins were only annotated in the Sanger database. In the remaining proteins, the observed differences were due to differences in annotation of transcriptional start sites.

**Conclusion:**

Our results indicate that, even in a less complex sample likely to represent only 10% of the bacterial proteome, we were still able to detect major differences between different gene annotation approaches. This gives hope that high-throughput proteomics techniques can be used to improve and validate gene annotations, and in particular for verification of high-throughput, automatic gene annotations.

## Background

Tuberculosis, the disease caused by the pathogen *Mycobacterium tuberculosis*, is responsible for approximately 2 million deaths annually according to the World Health Organization [1]. At the moment, the genomic annotation of several lineages of *Mycobacterium sp*. is available and largely validated [2-5]. However, the annotation of protein-coding genes is still a challenge in genomic sequencing projects despite advances in computational gene finding [6,7]. Consequently, differences in gene annotation introduced by diverse prediction methodology will have a major impact on subsequent studies. In addition, the presence of overlapping genes further increases the difficulty of annotation, resulting in theoretical protein products with different lengths.

An example of such differences can be observed from the number of Open Reading Frames (ORFs) annotated for the H37Rv laboratory strain of *M. tuberculosis *by two independent institutions [2,8], representing a difference of up to 12%, simply in the number of annotated genes. In addition, there are differences in the lengths of genes annotated by both institutions due to difference in start codon choice. Therefore, the validation of such annotations by identification of protein sequences is highly desirable to further refine the genomic annotations and enable generation of improved unbiased databases. Mass spectrometry based proteomic approaches (also referred to as "shotgun" proteomics) is by far one of the most sensitive, high-throughput methods available for large scale screening of peptides present in a particular sample (for a recent review, see [9]). Such techniques have emerged to become instrumental in proteomic projects aiming for systematic functional analyses of the genes uncovered by genome sequence initiatives [10]. Recently, MS-based approaches have been used to aid gene annotation in prokaryote and eukaryote genomes [11,12], and to validate genomic annotation. Deshayes *et al*. [13] demonstrated, for example, that a mass spectrometry driven validation could identify sequencing errors of the genome of *Mycobacterium smegmatis *that were mistakenly believed to be interrupted coding sequences.

The possibility for in-depth analysis of complex proteomes has been dramatically increased by recent developments in mass spectrometry-based proteomics [9], in particular, by a hybrid mass spectrometry Linear Ion Trap – Orbitrap mass spectrometer [14,15], in which ions are detected with high resolution by their motion in a spindle shaped electrode. It has recently been shown that, by using a 'lock mass strategy', very high mass accuracy is routinely achievable in both the MS and MS/MS modes [16], which virtually eliminates the problem of false positive peptide identification in proteomics.

In this article, we compared the revised original annotation for *M. tuberculosis *strain H37Rv from the Sanger Institute [2,17] with the annotation of the same sequence from the Institute for Genomic Research (TIGR) [8]. Previous results from our group suggested that differences in annotation may lead to divergent proteomic characterization [18], but such results were obtained using low-sensitivity, low-accuracy mass spectrometers. Therefore, we now generated a proteomic dataset from *M. tuberculosis *H37Rv culture filtrate acquired on a high-accuracy LTQ-Orbitrap instrument to improve identification coverage and reliability, and we specifically aimed to identify specific tryptic peptides represented in one or the other annotation.

Tryptic peptides specific to one or the other annotation can be observed when a complete gene is described in only one of the annotations. Specific tryptic peptides can also be observed when there is discrepancy in choice of the start codon of a particular gene. In that case, specific tryptic peptides can be seen in the N-terminal part of the longer gene. Correct choice of start codon may also be confirmed by observation of the very N-terminal peptide with or without its first methionine.

Our *M. tuberculosis *culture filtrates contain a high number of secreted proteins exported through the general secretory pathway [18]. In order to identify the N-terminal peptides of processed secreted proteins, where the signal peptide has been cleaved off, we used the SignalP algorithm [19,20] for identification of proteins with signal peptide, and the potential cleavage sites. Choice of start codon may however influence prediction of signal peptides using most signal prediction algorithms, including SignalP, because they consider the distance between the potential cleavage site and the precursor starting point. As a consequence, the N-terminal peptide of a mature secreted protein may not be detected if the choice of start codon precludes the prediction of the signal peptide [21].

We designed a database containing predicted N-terminal sequences in order to improve the identification of peptides in this area. To avoid repetitive entry generation and high levels of redundancy, the database was organized represented in a concise, MS-friendly format as described by Schandorff et al [22]. In order to determine the identification of single nucleotide polymorphisms (SNP) and N-terminal predictions, these authors created a modified IPI human database where the tryptic peptide containing a possible mutation was inserted at the end of the original entry, but always preceded by the letter "J" (representing no amino acid). Through this method they were able to test and identify several SNPs without compromising database size, redundancy and reliability of the results. Therefore, all gene entries in the Sanger and the TIGR annotations were submitted to SignalP v3.0 prediction, and sites with a sufficiently high score were selected and appended to the entries as described by [22].

In total, we were able to identify 449 proteins from the *M. tuberculosis *H37Rv culture filtrate fractions (comprising mainly extracellular proteins), representing a more in depth scale of identification from the previous study [18], a difference explainable solely on better MS instrumentation. From those, we detected and validated 35 peptides which were specific to one annotation, 34 of them were specific to the Sanger annotation and only 1 was specific to the TIGR annotation. In addition, the identified peptides resulted in the identification of 5 gene products whose genes are only annotated in the Sanger dataset (and not in the one from TIGR). These data represented 1.78% of all peptides identified in the study, comprising a rather small protein population detected, indicating that such observations could be even more critical with a larger dataset. Therefore, it is of significant importance to generate more precise, unbiased gene annotation datasets from *M. tuberculosis *to allow more efficient proteomic characterization.

## Results

### Proteomic description of the evaluated data

**Figure 1:**
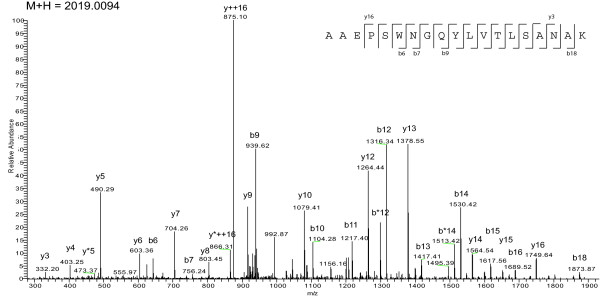
**MS/MS profile of ion M+H 2019.0094.** Tandem mass spectrum of a prevalent ion on a particular time point in the LC gradient and ionized on the LTQ-Orbitrap. The peptide fragments randomly on each amide bond, resulting in carboxy-terminal y ions or amino-terminal b ions. After the fragment masses were submitted to Mascot, the peptide was identified as AAEPSWNGQYLVTLSANAK (inset, with detected y and b ions represented) from protein Rv2253 – conserved hypothetical protein.

Protein samples from *M. tuberculosis *H37Rv culture filtrate were separated by 10% SDS-PAGE and each gel lane was excised in 10 fractions, as described previously by Målen et al [18]. Those bands were submitted to in-gel digestion and the resulting peptides were analyzed with a LTQ-Orbitrap mass spectrometer. Figure 1 shows an example of a MS/MS spectrum of a peptide of m/z M+H = 2019.0094. The Mascot engine identified this spectrum as the peptide AAEPSWNGQYLVTLSANAK, corresponding to the predicted N-terminal of the entry Rv2253/NT02MT2445 annotated as a ''conserved hypothetical protein'' (see Methods for details of N-terminal database prediction). Figure 1 also illustrates the fragmentation pattern and identification of y/b series on sequence (sequence input). In this example Mascot was able to correlate 14 of the possible 18 y ions for this sequence, and 12 of 18 b ions, resulting in an identification score of 128.

All data acquired from the LTQ-Orbitrap were analyzed using Mascot with two protein databases derived from Sanger respectively TIGR annotation of the *M. tuberculosis *H37Rv lab strain. In total, 449 different proteins were identified in this study. This is two times more proteins and almost four times as many peptides identified as compared to previous results from our group on the same culture filtrates [18]. Additional File 1 reports all peptides identified in this work, in addition to the protein entry to which each peptide belongs (only Rv from Sanger denomination were used to keep the analysis simple, an exception was done if the peptide belonged to the TIGR database, but not to Sanger). The table also contains information about protein mass, peptide length, observed charge, observed mass over charge ratio, measured peptide mass (Da) Mascot Score, the presence of modifications (as N-terminal acetylation or Met oxidation), the error of the observed/theoretical mass in ppm and a column showing the number of peptides per identified protein (see Additional file 1). Sequences in red indicate peptides which were identified in only one of the databases, as also indicated in Table 1.

**Table 1:** Specific tryptic peptides annotated in only one of the datasets

Accession number	Sequence	Peptide length	Measured mass [Da]	Score	Database	# Peptides/protein	ppm error	Left flank AA
Rv0063	VLQPDDGPQFATAK	14	1485.76	96	Sanger	23	2.4780	K
Rv0063	DPAASGWEALSSALGGK	17	1615.79	124	Sanger	23	3.4751	nterm
Rv0063	SGWEALSSALGGK	13	1261.64	86	Sanger	23	3.0629	nterm
Rv0063	GWEALSSALGGK	12	1174.61	73	Sanger	23	5.1922	nterm
Rv0363c	PIATPEVYAEMLGQAK	16	1716.89	64	Sanger	13	0.8069	
Rv0932c	ELHSSGSTAQENAMEQFVYAYVR	23	2632.21	82	Sanger	11	4.6848	K
Rv0932c	KELHSSGSTAQENAMEQFVYAYVR	24	2744.29	48	Sanger	11	0.3702	K
Rv3722	HQQDYAALQGMK	12	1388.66	78	TIGR	11	2.7728	R
Rv0928	ASGSTAQANAMTR	13	1264.59	84	Sanger	9	4.4435	K
Rv3874	AEMKTDAATLAQEAGNFER	19	2067.97	57	Sanger	9	4.6397	
Rv1437	SVANLKDLLAEGVSGR	16	1627.9	54	Sanger	8	5.6825	
Rv2889c	ANFTAADVKR	10	1091.58	65	Sanger	7	3.1234	
Rv0761c	IQMEAAGMCR	10	1181.51	53	Sanger	6	4.8286	K
Rv2430c	MHFEAYPPEVNSANIYAGPGPDSMLAAAR	29	3075.44	108	Sanger	6	3.1084	
Rv2290	SYTLTGTGHAVIPGQTGMR	19	1945.98	104	Sanger	5	1.8289	K
Rv2290	IGSVDYQMPYQPVQSPTQVEATR	23	2609.26	84	Sanger	5	4.0165	K
Rv2290	VNAHDDSASVTLSLSDSTPPDVNGFGISLK	30	3042.49	71	Sanger	5	3.1941	R
Rv2290	ELPFGVHVTCP	11	1254.62	41	Sanger	5	3.0884	R
Rv2290	SYTLTGTGHAVIPGQTGMR	19	1961.98	35	Sanger	5	4.8758	K
Rv3705c	HPSEPGVVSYAVLGK	15	1538.82	84	Sanger	5	2.0407	nterm
Rv3705c	SEPGVVSYAVLGK	13	1304.71	73	Sanger	5	5.3952	nterm
Rv0753c	SADVFDPNTGQIQAK	15	1589.78	87	Sanger	4	2.3470	R
Rv0753c	TTQISHFIDGQR	12	1401.71	64	Sanger	4	4.5361	
Rv0321	LGIDPFDDTLVQPSSIDVR	19	2086.07	80	Sanger	3	3.0416	R
Rv0549c	ASPTSPPEQVVVDASAMVDLLAR	23	2352.22	41	Sanger	2	4.8829	R
Rv1352	TEGPSNPLILVFGR	14	1498.83	64	Sanger	2	2.8161	R
Rv1352	ETGEQFPGDGVFLVGTDIAPGTYR	24	2525.22	100	Sanger	2	0.7501	nterm
Rv1810	DYNPGLTMDSAAK	13	1381.63	78	Sanger	2	4.0868	R
Rv1810	FAAIASGAYCPEHLEHHPS	19	2092.96	43	Sanger	2	4.6181	K
Rv3800c	AELTVPEMR	9	1044.54	28	Sanger	2	3.3181	R
Rv0500	IAIIGGGSIGEALLSGLLR	19	1809.08	118	Sanger	1	2.5894	R
Rv2752c	VTALGGINEIGR	12	1198.68	92	Sanger	1	2.9576	R
Rv3624c	SVLLTAEQIQAR	12	1327.75	75	Sanger	1	2.3044	K
Rv2035	FTAQSAETTR	10	1110.54	49	Sanger	1	3.5484	R
Rv1987	SGTHYVLSPANWNR	14	1600.79	48	Sanger	1	9.8228	R

Since our objective in this study was to scrutinize the differences between the databases, and not to generate a catalogative dataset, we allowed the validation of proteins identified with 1 peptide. While such criteria is rarely employed in proteomic studies, we argue that these peptides should be included in our analysis since they have been identified with a highly accurate instrument in an organism with a small genome and thus represent highly statistically significant results. Therefore, the identified proteins in the Additional File 1 were divided in 3 categories. First, all proteins with at least 2 peptides with minimal score of 21 (representing a p-values less than 0.01 per peptide according to Mascot), followed by proteins with only 1 peptide but Mascot score higher than 42 (false-positive rate of 1/10000), and finally, 23 proteins with 1 peptide with score between 30 and 42 (false-positive rate of 5/10000). Using such score criteria, we had no Reversed hits being validated in the analysis (all were identified with only 1 peptide and score lower than 26 – Data not shown), confirming that our dataset should have extremely low false-positive rates. However, it is important to note that we performed such category separation solely to help the reader to discriminate such identifications as lower confidence ones, and they are statistically acceptable.

### Sanger versus TIGR annotation comparison

The initial comparison of Sanger and TIGR gene annotation datasets were mostly based on the fact that we were previously able to identify three proteins that were specific to one dataset [18]. In addition, there are several differences in the annotations which are available at the TIGR Comprehensive Microbial Resource website [8]. Therefore, we decided to create a comprehensive list of genes annotated on each of the two datasets, and also to determine the genes specific to each. Our alignment was based on genes that shared the same stop codon, independent of having different start codons. Additional File 2 shows a list of 3750 genes that share the same stop codon in both annotations. This table contains an alignment of the similar annotated genes of both datasets, indicating its position in the genome, the DNA gene size, the protein product size, GC content ratio, the Stop Codon (StopC) for the gene, and if available the Swiss-Prot code. Finally, we also calculated the difference in length of the equivalent annotated protein products (see Additional file 2).

**Figure 2:**
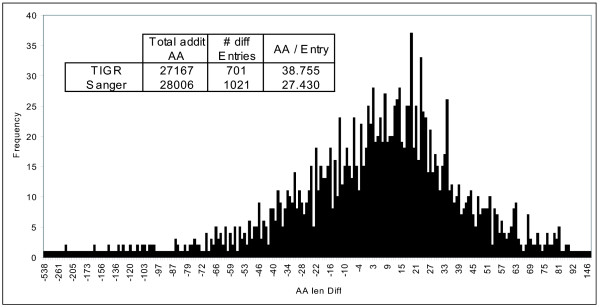
**Length comparison between genes annotated in both Sanger and TIGR datasets.** When the TIGR and Sanger datasets where compared, 46% of the genes present on both sets differed by chosen TSS. The graph shows frequency distribution (number of genes) and number of amino acid difference on the N-terminal side (AA len diff). While the distribution by number of cases is higher in the Sanger dataset (1021 genes with longer products compared to the same gene in TIGR, inset Table), genes that are longer on TIGR tend to be exceedingly longer when compared to Sanger.

From those, 2025 had exactly the same base pair/protein sequence size, while 1725 (46%) had different start codons in the two datasets. Strikingly, there are some extremely divergent annotations in terms of gene size, as can be seen for the TIGR entries NT02MT1622 (840 amino acids,) or NT02MT0741 (401 amino acids), which are 538 and 259 amino acids longer than the respective entries, Rv1483c and Rv0677 in the Sanger annotation. However, most of the differences are within the range 10 to 15% of the total sequence length. Figure 2 illustrates the length distribution of the protein product of these similarly annotated genes, and it is clearly observable that the differences are equally distributed between datasets, i.e., both TIGR and Sanger contribute similarly to the differences observed, therefore initially eliminating the possibility that one of the datasets were generated through a less stringent methodology. For example, while TIGR has 701 entries with longer genes, Sanger has a slightly higher frequency of such cases, with 1021 entries. However, the ratio of exclusive amino acid sequences per entry is higher in TIGR (38 amino acids) compared to Sanger (27 amino acids) (Table Inset, Figure 2).

### N-terminal prediction and database generation

Since most of the observable differences belong to the N-terminal side of the proteins, and based on the knowledge that exported proteins from *M. tuberculosis *can be submitted to N-terminal processing by a signal peptidase complex [23], we were naturally concerned that a possible lack of capability to identify such differences could be a result from a database limitation to generate tryptic peptides which correspond to the post-processed N-terminal of the protein. This was already described by Rison et al. [24], who generated theoretical tryptic peptides to identify the correct TSS of 11 proteins from *M. tuberculosis*. However, those authors chose a database setup which was redundant (each new protein version has a new entry). This limitation was satisfactorily solved by Schandorff et al [22], where the authors designed a database where ''artificial'' tryptic peptides representing predicted N-terminal sequences from human proteins were inserted at the end of its respective protein entry, consequently allowing the identification of this peptide by mass-spectrometry.

**Figure 3:**
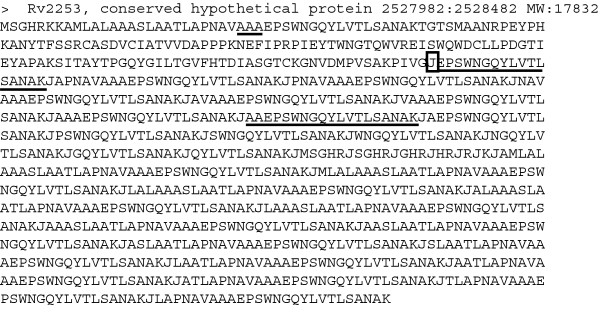
**Example of MS-friendly database entry for N-terminal prediction validation.** This entry represents the protein Rv2253 (Conserved hypothetical protein) which is 167 amino acids long. Analysis with the tool SignalP v3.0 resulted in the prediction of the sequence A27-A28-A29 (underlined) as a possible cleavage site of signal peptidase I. Therefore, the predicted N-terminal peptide is inserted after a J (box). In addition, we also appended all peptides possible from position -25 until +7 from the predicted signal peptidase cleavage site. In this case, we not only identified the predicted N-terminal peptide starting in E30 (underlined after box) but also a second peptide starting on amino acid A28 (last underline – see Figure 1 for MS/MS data) representing a possible N-terminal alternative option.

To better identify peptides from the N-terminal region of the entries, we designed a similar MS-friendly database as cited above, where all entries that had a signal peptide predicted by the SignalP tool v3.0 [19,20] were modified to insert the post-processed sequence as a tryptic peptide J-tagged to the original sequence. This method is illustrated in Figure 3. In this example, the protein Rv2253 from the Sanger dataset had the N-terminal predicted to start in position E30, just after the sequence portion AAA, confirming an AXA motif (marked with a short underline). While the peptide EPSWNGQYLVTLSANAK is not originally a tryptic peptide in the entry (as it is preceded by an A, not an R or K), its chance of successful identification is non-existent. Therefore, this peptide is re-inserted in the entry after a J letter (box) and Mascot trypsin parameters are changed to consider the J code as a cleavage site of trypsin.

We also considered that additional cleavage sites could be preferred in the neighboring predicted site, or that the prediction was incorrect. Therefore, as shown in Figure 3, we also inserted in the entry all possible tryptic peptides from the position -25 up to +7 of the predicted site, to achieve a better coverage of possible positions of N-terminal cleavage. In this study, we utilized this method on both the TIGR and Sanger datasets, in order to obtain a better chance to identify any exclusive peptides on the N-terminal side of the entries. A total of 44 peptides from 32 different proteins were identified and are listed in Additional File 1 and marked as ''Nterm'' on its left flanking amino acid category. From those 44 peptides, six peptides from proteins Rv0063, Rv3705c and Rv1352 are specific to one dataset. Interestingly, for Rv2253 (Figure 3), we identified both the predicted N-terminal in E30, as well as an additional sequence starting in A28 (see Figure 1 for MS/MS) (both peptides underlined in Figure 3). Interestingly, the amino acid A28 is also following an AXA motif, suggesting that in this case Signal Peptidase I may recognize and cleave at different portions of the same molecule.

### Specific tryptic peptides on Sanger or TIGR identified by LC-MS/MS

Once we generated the peptide lists of the identified proteins from Sanger or TIGR databases, we compared them in order to determine the presence of peptides or proteins which are specific to one of the datasets. To characterize a peptide as such, it is relevant to point out that the observed sequence should be absent from similar entries representing the same gene, and should also be absent from other entries in the database or from the reversed sequences inserted as false-positive delimiters.

In total, we identified 35 peptides which were specific to one of the databases, representing a total of 24 different proteins (Table 1). This table is a summarized version of Additional File 1 showing only the specific peptides, their sequences, their Mascot Score, total number of peptides identified for that protein hit (including peptides that are shared in both databases), in which database they were observed and finally the ppm error observed for the parental ion. While 30 of those specific-dataset peptides were identified for protein hits in conjunction with other peptides (indicating that the gene product detection is correct), 5 of these peptides were observed given our identification criteria of "1 peptide per hit". However, those peptides had still a very high Mascot score (ranging from 48 for Rv1987 and Rv2035 to 128 for Rv0500). In addition, the *in silico *trypsinization of the databases performed by Mascot could not identify any other sequence-combinations that could fit with the observed spectra, when such a small mass error as 10 ppm was required on the database search (data not shown).

**Figure 4:**
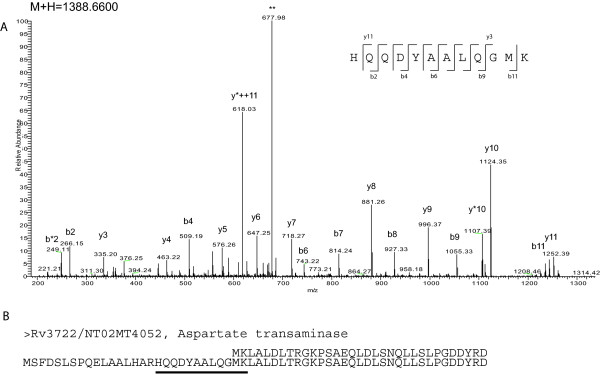
**A specific tryptic peptide observed in the TIGR annotation.** In total, we identified 35 peptides which were specific to Sanger or TIGR datasets. This figure illustrates the only example observed only in the TIGR database. In (A), MS/MS information of the ion M+H = 1388.6600. While this MS/MS spectrum could not be identified by Mascot when using the Sanger database, it was identified as sequence HQQDYAALQGMK (inset with fragmentation pattern) only when the TIGR database was used. When the N-terminal region of this entry was aligned with the corresponding gene annotated by Sanger Rv3722 (B), it is clear that the Sanger entry (Top) failed to annotate the correct TSS for this gene. The identified sequence is underlined in the TIGR entry (bottom).

Surprisingly, the vast majority of specific peptides were observed in the Sanger database, while only one peptide, from the protein Rv3722/NT02MT4052 was specific to the TIGR database (Figure 4). In this figure, it is possible to observe the CID fragmentation pattern of the peptide HQQDYAALQGMK (mass of 1388.66 Da) from the protein Aspartate transaminase, identified with a Mascot score of 78. This peptide had 9/11 theoretical y ions detected, and 7/11 b ions. Below the MS/MS spectra, the alignment between the Sanger (top) and TIGR (below) annotation is shown, indicating that the identified sequence was only annotated as part of the Aspartate transaminase gene in the TIGR dataset (underlined).

**Figure 5:**
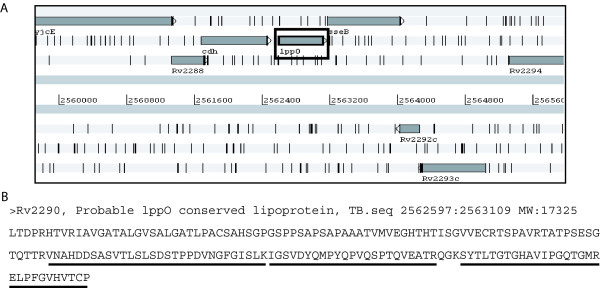
**Identification of specific protein products.** From the 35 specific tryptic peptides reported, we were able to identify 5 proteins that were only annotated in the Sanger database. The protein Rv2290 is an example of this. The visualization of this gene in the genome using Artemis tool and Sanger annotation (box) is illustrated in (A), while the same genomic region does not contain any annotated gene in TIGR (not shown). The sequence of this protein is shown in (B), which was identified with four tryptic peptides. The sequence of these peptides is represented with underlining.

Most of the differences were due to differences in N-terminal annotations, but 5 of these 24 proteins were also specific to Sanger, while their peptides and the corresponding genes were not annotated in the TIGR database. Figure 5 represents one of these proteins, Rv2290, a probable lppO conserved lipoprotein, visualized using the Artemis tool and the Sanger genomic database [25]. The genomic region containing lppO, marked with a square, has no defined gene annotation according to the TIGR database (data not shown). Below the Artemis visualization, the sequence of the protein is represented, and the sequences of the identified peptides (4 in total plus 1 of them were also identified with an oxidized methionine – see Additional File 1) are shown underlined.

## Discussion

In prokaryotes, due to its simpler gene structure (i.e., lacking intron/exon organization), ORF determination using a combination of bioinformatic tools such as coding potential and homology to other confirmed genes from other organisms, is a relatively efficient task, which is achieved through automated and, in lower scale, manual curation (see [26] for a recent review). However, high-throughput genomic sequencing, and consequently automatically generated ORF determination, results in the identification of genes or gene families which are purely hypothetical. Therefore, the determination of the correct coding sequence regions, and its validation, are still challenging tasks. This is illustrated, for example, with the existent difficulty to predict the translational starting site of the predicted ORF. Our results show that, even with the constant advances in the *in silico *analysis of genomes and in the annotation of ORFs, there are still difficulties to establish reliable determination of coding sequence regions. Proteomic approaches are a powerful method to validate such annotations. Using two dimensional electrophoresis, Jungblut *et al*. [27], demonstrated this through the identification of protein products from genes which were not reported on the first gene annotation of *M. tuberculosis *H37Rv performed at the Sanger Institute [2].

It is important to note that differences in annotations as observed for Sanger and TIGR are expected, due to differences in the methodology used for predicting and validating coding sequences [28,29]. In addition, since primary annotations have been available for a longer time and tested more extensively (as exemplified above by [27]), they are probably more refined, as is the case for the Sanger *M. tuberculosis *H37Rv dataset. However, an extensive generation of protein databases through different prediction methods, without the concern to integrate them, can result in less efficient proteomic characterization on further studies. It is then relevant to be able to validate, through peptide identification, which gene is appropriately annotated, in order to provide more concise and unbiased databases.

It is striking that the TIGR dataset, even though it includes a higher number of predicted genes, still failed to annotate 5 proteins (Rv2290, Rv1352, Rv1810, Rv1987, Rv2035). Similar results were already observed when unpredicted protein products of lipoproteins from *Methylococcus capsulatus *were identified [30]. While our results give a straightforward indication that the annotation criteria produced by the group at the Sanger Institute is more reliable than the TIGR, we do not exclude the possibility that this observation may be due to the fact that we performed our validation on a sample that represents only a small portion of the *M. tuberculosis *proteome. For example, we were able to identify slight differences as observed for ESAT-6 (Rv3874), one of the main components of *M. tuberculosis *exported proteins [31] and only 3 amino acids longer in the Sanger database, but failed to detect several other proteins with more remarkable differences. However, it is still important to point out that further annotation comparisons using TIGR datasets and other primary annotations could be performed in order to establish more reliable datasets. The Comprehensive Microbial Resource has, at the moment, 401 total genomes mostly sequenced by other institutes/consortiums. In some cases, differences in annotations can have variations as extreme as that observed for the organism *Mycobacterium leprae*, with a primary annotation of 2720 genes [3], but with a TIGR annotation of 5398 protein coding genes.

Another important advantage of comparing databases through such an approach is to determine the translational start site (TSS) of the annotated gene. This is relevant not only to define the amino acid sequence of the protein (since the stop codon in unambiguous) but, most importantly, to define the upstream region of the ORF itself. Genome-wide and focused studies of promoter structure and other regulatory motifs depend on the correct definition of the intergenic regions defined by the coding sequence annotations [32,33]. Therefore, a correct TSS assignment directly affects the analysis of both protein function and transcriptional regulation. For *M. tuberculosis*, it was shown through a proteomic approach [24] that the protein Rv1099c had an incorrect TSS, and that reassignment completely altered the predicted promoter region for the gene. While in our study we can not ensure that the specific peptides are indeed the N-terminal of the protein, our results are still valid in order to eliminate the incorrect TSS annotated in one or the other dataset.

Furthermore, differences in TSS choices of the annotated gene will also influence subsequent *in silico *analysis, like N-terminal predictions used in this article. For example, we observed two cases (entries Rv0519c and Rv3484) where the cleavage site for signal peptidase I was correctly assigned only in the TIGR dataset, even though the region containing it is correctly annotated on both datasets. However, both entries are shorter in the TIGR database (15 amino acids for Rv0519c and 38 amino acids for Rv3484), meaning that the predicted cutting site is closer to the TSS choice of TIGR than it is in the Sanger entry, resulting in higher score results for its correct prediction.

**Figure 6:**
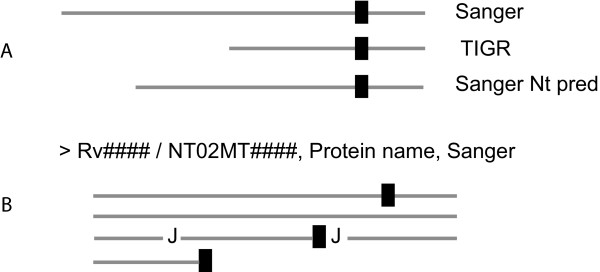
**MS-friendly database generation as a solution to discrepancy of datasets.** As it was reported by Schandorff et al. [22], we propose the creation of a unified database where differences in the N-terminal side of annotated genes can be easily accommodated to improve proteomic identification. In this example, the N-terminal of a gene annotated in Sanger, TIGR and a predicted cleavage site of the Sanger are considered (A). The alignment in (A) only shows the N-terminal region to facilitate comparison, with the first common tryptic site as a black box. When the entry is generated, the sequence of the longer version is kept (in this case, Sanger). Only the tryptic peptides comprising the TIGR N-terminal and the Sanger predicted N-terminal are inserted after a J. Such an approach not only allows the identification of all sequence variations within a single and simplified entry, but also eliminates redundancy from regions where the annotated sequences are identical.

Therefore, it is desirable to create more concise databases with as little redundancy as possible. Differences in annotations from different institutes or consortiums can be assimilated as a single file, allowing a more precise analysis through proteomic approaches. A perfect example of this was demonstrated by [22] and their MS-friendly database, where they not only created modified entries to confirm N-terminal predictions, but also to validate cSNP variations and sequence disagreement between different databases. While in most of their cases the difference resides in only one amino acid from case to case, the same method could be easily used to create concise databases for *M. tuberculosis *or even for other bacteria. Figure 6 shows an example of how such a database could be built. In this case, the longer entry (Sanger) would be kept as the "original" entry in the new database. The shorter TIGR version, with an N-terminal part that could not be identified as a tryptic peptide in the Sanger version is inserted at the end of the "original" entry separated by a neutral code letter. Additional N-terminal or TSS predictions from both Sanger and TIGR could also be inserted in the final file. Such an approach would probably increase identification efficiency considerably in following proteomic studies.

## Conclusion

By using a combination of high-accuracy mass spectrometry data acquisition, N-terminal prediction and gene annotation comparison, we were able to identify 35 tryptic peptides which were specific to one or other of the two most used databases of the *M. tuberculosis *H37Rv laboratory strain. Our data indicates that even on a less complex sample isolated from culture filtrate (representing close to 10% of the total predicted proteome), such differences in gene annotation may be a limitation to proteomic identifications, since specific proteins for one of the databases were also detected. We propose that a more concise and efficient database generation, including divergent annotation, should be achieved to improve MS-based identifications as recently demonstrated with human cSNP validation.

## Methods

### Bacterial culture and sample preparation

*M. tuberculosis *H37Rv ATCC27294 strain from the National Institute of Health (Tokyo, Japan) was cultured as surface pellicle on the wholly synthetic Sauton medium for 3 weeks without shaking. Bacteria were removed by filtration and the culture filtrate was concentrated by 80% ammonium sulphate precipitation. Precipitated proteins were dissolved in buffer and dialyzed against distilled water and lyophilized [34].

### SDS-PAGE and in situ digestion

Fifty micrograms of *M. tuberculosis *H37Rv culture filtrate proteins were diluted in 15 μL of deionized water, mixed with 5 μL of eletrophoretic sample buffer (NuPAGE kit, Invitrogen, Carlsbad CA, USA) with 1 μL DTT 100 mM, and boiled for 5 min at 56°C prior to electrophoretic run. Proteins were separated using a NuPage 10% Bis Tris Gel (Invitrogen) in MES buffering system at 200 V constant voltage. The sample migration was allowed to proceed until the blue dye reached the bottom of the gel. The proteins were further visualized using a Colloidal Coomassie Novex kit (Invitrogen).

After staining, each gel lane was divided in 10 fractions as described by [18], sliced in minor pieces and submitted to *in gel *reduction, alkylation and tryptic digestion. Proteins were reduced using 10 mM DTT for 1 hour at 56°C and alkylated with 55 mM iodoacetamide for 45 minutes at room temperature. The reduced and alkylated peptides were digested with trypsin 1:50 wt:wt (Sequence grade-modified, Promega, Madinson WI, USA) for 16 hours at 37°C in 50 mM NH_4_HCO_3_, pH 8.0. The reaction was quenched through acidification with 2% trifluoracetic acid (Fluka, Buchs, Switzerland). The resulting peptide mixture were desalted on RP-C_18 _STAGE tips [35] and diluted in 0.1% trifluoracetic acid for nano-HPLC-MS analysis.

### N-terminal Database

To predict signal peptide cut sites, we used the standalone version of SignalP 3.0 [19,36,20]. This program uses Neural Network (NN) and Hidden Markov Model (HMM) methods to predict likely sites based on data known from the literature. The program may predict one cleavage site by the HMM method and up to three using the NN method which often, but not always, is the same. The HMM method has a tendency to print out a specific site to have a probability of 0, so we removed HMM predictions with a probability less than 0.25. The remaining predictions appear to include the true predictions. In this work, we used SignalP with the training data for gram positive bacteria.

We prepared two databases of the *M. tuberculosis *dataset, one based on the primary (Sanger) annotation [17] and one based on the TIGR annotation [8]. The Sanger database had 4010 sequences, and SignalP predicted at least one signal peptide cut for 1315 of them. The TIGR sequences had 4219 entries, and of these 1350 had at least one prediction. Considering that the prediction methodology is not completely accurate, we also considered that the cut site could occur in the neighborhood of a predicted site. Therefore, we tested all N-terminal possibilities starting 25 amino acids upstream until 7 downstream of the predicted site (see Figure 3), until the first tryptic site is found. Each of those peptides were appended at the end of the protein sequence, each separated by the letter "J" [22]. The Mascot search engine was then configured to recognize "J" as a possible tryptic site. In addition, we also included in the database the reverse of each of the original entries for false-positive identification control of the proteomic data [37]. The whole process including running SignalP was automated using an in-house program.

### Mass Spectrometry

All experiments were performed on a Dionex Ultimate 3000 nano-LC system (Sunnyvale CA, USA) connected to a linear quadrupole ion trap – Orbitrap (LTQ-Orbitrap) mass spectrometer (ThermoElectron, Bremen, Germany) equipped with a nanoelectrospray ion source (Proxeon Byosystems, Odense, Denmark). For liquid chromatography separation, we used a capilar of 5 cm bed length 100 micron ID self packed with Reprosil_Pur C18-aq (Dr. Maisch Gmbh, Ammerbuch-Entringen, Germany). The flow rate used was 0.2 μL/min for the nano column, and the solvent gradient used was 5% B to 60% B in 42 minutes, then from 60% B to 95% B in 10 minutes. Solvent A was aqueous 2% acetonitrile (ACN) in 0.1% trifluoracetic acid, whereas solvent B was aqueous 80% ACN in 0.1% trifluoracetic acid.

The mass spectrometer was operated in the data-dependent mode to automatically switch between Orbitrap-MS and LTQ-MS/MS acquisition. Survey full scan MS spectra (from m/z 400 to 2,000) were acquired in the Orbitrap with resolution R = 60,000 at m/z 400 (after accumulation to a target of 1,000,000 charges in the LTQ). The method used allowed sequential isolation of the most intense ions (up to five, depending on signal intensity) for fragmentation on the linear ion trap using collisionally induced dissociation at a target value of 100,000 charges.

For accurate mass measurements the lock mass option was enabled in MS mode and the polydimethyilcyclosiloxane (PCM) ions generated in the electrospray process from ambient air (protonated (Si(CH_3_)_2_O)6; m/z 445.120025) were used for internal recalibration during the analysis [16]. Target ions already selected for MS/MS were dynamically excluded for 30 seconds. General mass spectrometry conditions were: electrospray voltage, 1.9 kV; no sheath and auxiliary gas flow. Ion selection threshold was 500 counts for MS/MS, an activation Q-value of 0.25 and activation time of 30 ms was also applied for MS/MS.

### Mascot search and peptide/protein validation

Protein identification was performed by searching the data separately against the databases (including alternative N-termini as described above) derived from the Sanger and TIGR annotations, using MASCOT Deamon (Matrix Science). The search parameters used were: Maximum missed cleavages: 3; Carbamidomethyl (C) as fixed modification; N-acetyl (Protein) and Oxidation (M) as variable modifications. Peptide mass tolerance of ± 10 parts per million; MS/MS mass tolerance of 0.5 Da. Under such criteria, Mascot indicated a minimal score of 21 for p = 0.01 and 15 for p = 0.05, in both Sanger and TIGR datasets. All data had a mass accuracy average of 3.8 parts per million. Spectra and protein validation were performed using an open source software called MSQuant, largely used for LC-MS/MS data analysis [38]. Proteins were validated accordingly in three categories (see Additional File 1): those with at least two, fully tryptic peptides with a minimal score of 21 for individual peptides; those with only 1 peptide, but individual MS/MS score higher than 42 (p = 0.0001); and finally those with score higher than 30 (p = 0.0025). Under such criteria, all MS/MS identifications of peptides present in entries with reversed sequences (i.e., false-positive identifications) were not validated, since none of them were identified with 2 peptides with score higher than 21 each or 1 peptide with score higher than 30.

## Authors' contributions

GAdS contributed to overall conception and design, analysis and interpretation of data, and manuscript drafting. HM and TS contributed with proteomic data acquisition and critical revision of manuscript. GS designed the script for N-terminal database generation after SignalP prediction. SP contributed with data analysis and interpretation of genomic data. IJ contributed to conception and design and critical revision of the manuscript. HGW contributed with conception and design, project coordination, manuscript drafting and critical revision. All authors had read and approved the final manuscript.

## Supplementary Material

Additional file 1 List of identified peptides. The file reports all peptides identified by proteomics using a LTQ-Orbitrap mass spectrometry. Click here for file

Additional file 2 Comparison between genes annotated for H37Rv. The file contains comparisons of Locus from *M. tuberculosis *H37Rv that were similarly annotated between Sanger and TIGR institutes. Click here for file
